# Reading Aloud

**Published:** 1874-07

**Authors:** 


### READING ALOUD.

The first requisite of an orator is such a distinctness of enunciation that every word he says can be clearly heard without an effort.

It is not by any means necessary that he should speak loud, for then there is such a resonance of the voice, such a rebounding of sound, as to interfere with distinctness of speech. Persons who are partially deaf hear those best who speak to them in a soft voice only, if the enunciation is clear and well defined. Bellowing is not speaking. Nothing better trains a speaker's ear than to accustom himself to read aloud in conversational tones. Young men who contemplate public speaking cannot do better than read thus, standing or walking, the breast slightly protuberant; this may be done half an hour at a time, and repeated three or four times a day. Several important advantages would result from such a practice almost infallibly.

1. It aids in developing the capacity of the lungs for taking in a larger amount of air.

2. It habituates the voice to public speaking, and gives it not only greater volume but greater power, without exciting a tickling in the throat or hemming or coughing.

3. It habituates the ear of the reader to the tone, rhythm of language, and to a smooth, grammatical expression. Bad grammar may be detected by the ear, without the intellect being capable of pointing out the faultiness of construction.

4. If the reading is performed peripatetically in the open air, it is very greatly promotive of health, for every student should make it an imperative rule to obtain for himself an out door exposure of not less than two hours of the twenty-four, as a means of keeping up a healthful circulation of the blood, and the purification of it consequent on breathing an atmosphere free from all contaminations.

5. One of the very best means of composing a speech and of expanding a text or subject, is to speak upon it in the open air, under a kind of impression that a large audience is present; this exalts the imagination—under the influence of which the subject expands, new ideas and illustrations present themselves which were not in view at the commencement of the exercise; just as when we get a little excited in writing a letter or penning a composition, forms of expression come up which we little dream of at the commencement. The most uncultivated find little difficulty in writing a letter after having started.

